# Cost Analysis of Postoperative Medications in a Tertiary Care Hospital in Northeast India

**DOI:** 10.7759/cureus.81688

**Published:** 2025-04-04

**Authors:** Indrani Sarma, Sukainnya Buragohain, Joonmoni Lahon, Priyotosh Banerjee, Chayna Sarkar, Noor Topno, Dibyajyoti Saikia, Dhriti K Brahma

**Affiliations:** 1 Pharmacology, All India Institute of Medical Sciences, Guwahati, Guwahati, IND; 2 Pharmacology, ICARE Institute of Medical Sciences and Research and Dr. Bidhan Chandra Roy Hospital, Haldia, Haldia, IND; 3 Pharmacology, North Eastern Indira Gandhi Regional Institute of Health and Medical Sciences, Shillong, IND; 4 General Surgery, North Eastern Indira Gandhi Regional Institute of Health and Medical Sciences, Shillong, IND

**Keywords:** antibiotic prophylaxis, cost analysis, cost simulation, elective surgery, postoperative complications, postoperative medications

## Abstract

Background: Postoperative medications play a crucial role in managing physiological changes and preventing complications after surgery. However, the cost of these medications can be a significant burden for patients, particularly in low-income settings. This study aimed to estimate the cost of postoperative medications in a tertiary care hospital and simulate the financial burden on patients undergoing elective surgery.

Methods: A prospective, hospital-based observational study was conducted at a tertiary care hospital in India. Data were collected from 109 patients undergoing elective surgery, and the cost of medications was calculated using real-world data. A gamma distribution was used to simulate 100000 data points, and the mean, median, and interquartile range of medication costs were estimated.

Results: The mean medication cost was ₹9332 (around $111 USD), with a median cost of ₹4989 (around $60 USD). The interquartile range was ₹11103, indicating substantial variability in patient costs. The simulation results showed that patients would need to work for an average of 51 days to cover the mean cost of medications, with up to 104 days required for 2.5% of patients.

Conclusion: The financial burden of postoperative medications is significant. The findings highlight the need for flexibility in government schemes like Ayushman Bharat to accommodate the variability in costs. Policymakers should consider revising the sanctioned amounts per treatment module to keep pace with inflation and changes in medical practice.

## Introduction

Postoperative medications are critical in managing the physiological changes and potential complications that occur after surgery. Pain management is often the primary focus, with opioids, nonsteroidal anti-inflammatory drugs (NSAIDs), and acetaminophen used to control varying levels of pain [1]. Antibiotics are commonly administered to prevent or treat infections, particularly in surgeries with a high risk of contamination [2]. Surgical site infections (SSIs) present a significant challenge to patient recovery and healthcare costs, leading to complications, prolonged hospital stays, and additional treatments [3]. The incidence of SSIs is estimated to range from 3% to 50%, depending on the type of surgery [4,5]. The management of SSIs, including extended care and medications, further adds to surgical expenses, underscoring the importance of addressing SSIs to improve patient outcomes and reduce overall healthcare costs.

Beyond pain and infection control, other medications address specific postoperative challenges. Antiemetics, such as serotonin receptor antagonists and dopamine antagonists, are used to prevent nausea and vomiting [6], while proton pump inhibitors (PPIs) and H2 receptor antagonists help reduce gastric acid secretion, preventing stress ulcers [7].

The selection of postoperative medications is tailored to the patient’s needs and the type of surgery performed to optimize recovery, minimize complications, and ensure patient comfort. The proper administration of these medications is essential for enhancing postoperative outcomes and facilitating a smooth recovery process.

To advance universal health coverage (UHC), the Indian government provides annual coverage up to ₹500000 under Ayushman Bharat Pradhan Mantri-Jan Aarogya Yojana (AB PM-JAY), which was launched in September 2018 [8]. Approximately 60% of the health benefit packages (HBPs) under this scheme are for conditions requiring surgical procedures. The scheme currently covers 1949 surgical and medical procedures. The coverage amount was set based on the "Costing of Healthcare Services in India" (CHSI) study [9].

The primary objective of the study was to estimate the cost of postoperative medications for general surgical procedures during hospital stay. This study presents results from a simulation study using real-world prospective data collected from a tertiary care center and reports the cost of postoperative medications used by a bottom-up approach. It provides valuable insights into the costs of surgical care in government tertiary hospitals, offering essential information for revising reimbursement rates for HBPs. The secondary objective of this study was the assessment of the pattern of antimicrobial usage. 

## Materials and methods

The study was conducted at the North Eastern Indira Gandhi Regional Institute of Health and Medical Sciences (NEIGRIHMS) in Shillong, India, a tertiary care hospital. It involved patients admitted to the general surgery ward from May 2021 to April 2022. The study was approved by the Institutional Ethics Committee of NEIGRIHMS on April 26, 2021, under the approval number NEIGR/IEC/M14/T25/2021. The study was designed as a prospective, hospital-based observational study where enrolled participants were followed up from admission till discharge. Data were collected from inpatient progress records, patient history records, anesthesia records, operation records, preoperative checklists, treatment charts, and nursing records. The data reflected the use of medications that were prescribed by the Department of General Surgery for the participants in the postoperative period. The study included adult patients from 18 to 65 years of age who were admitted for routine surgical procedures in the general surgery department. Exclusions were patients from other surgical departments, those with pre-existing infections or admitted to the intensive care unit, those undergoing minor procedures, or those unwilling to give consent. Demographic details, medical history, and treatment information were collected in a pre-validated standard participant data record form.

The treatment given is protocol and guidelines-based and seldom affected by the paying capacity or insurance coverage of the patients. The costs calculated are the costs of the medication if bought from the market, irrespective of whether the patient paid or was subsidized, or paid from insurance or any government-run health coverage program. This study did not examine out-of-pocket expenditure. The costs calculated were for the whole duration of stay in the hospital. Medications prescribed at the time of discharge were not included in the analysis. This was done as the objective of our study was to find the cost of medications used during a hospital stay postoperatively and not the total cost of treatment. No discount rate was applied as medications were used for a short duration only, and the endpoint of data collection was discharge from the hospital.

The cost of medication was calculated from a market survey. The survey was designed to determine the prevailing market prices (maximum retail price) of administered medications of different brands across various retail outlets in the geographical area. A data collection form was developed to capture information on the unit price of each medication, along with formulation details (such as dosage and packaging size) and the unit and total prices. Any vendor-specific discounts on the maximum retail price were not considered for the evaluation in the study. Only labeled prices were included. The median market price was used to calculate costs. Median prices have been used by various studies previously to analyze drug prices [10-12]. The total cost of each medication during the hospital stay of each participant was used in the analysis. The medications were categorized into four groups: antibiotics, medications for postoperative pain relief, nutritional support, and others. Others included drugs used for vomiting, control of bleeding, prevention of peptic ulcers, and other medical causes specific to each patient. Costs were calculated in Indian currency (Indian rupees) and compared with the government-mandated minimum daily wage for unskilled workers. Costs are for the year 2022-2023 [13].

Raw patient data from the real world was used in our statistical model. The model was designed to estimate the mean, median, and quantiles of postoperative medical costs to assess what proportion of the population will have how much cost. Data from patients were used to model the outcomes. The model was used to simulate 100000 patients to estimate costs in the target population from a sample group of patients. Individual patient-level data were used to generate the characteristics of the statistical distribution of costs, which was used as input in the simulation model. Simulated runs were made with this distribution for stochastic evaluation. Parameter uncertainty (in costs) was propagated through the model as it allowed the values to vary randomly according to the specified distribution.

We used the gamma distribution to simulate costs in our model. The gamma distribution has been reported to be better in simulating costs compared to other distributions [14]. The gamma distribution can handle skewed cost data, which is common in real-world scenarios, where extreme values can occur [15]. The gamma distribution is a conjugate prior for various likelihood functions, facilitating Bayesian inference and uncertainty quantification in cost estimation [16]. The gamma distribution can model costs with varying degrees of variability.

Gamma distribution simulation

R software (Version 4.4.0, 2024-04-24 ucrt, "Puppy Cup") 16 was used to simulate 100000 data points of total cost. The shape parameter and rate parameter (α and β) for the gamma distribution were calculated using the mean (₹9175) and standard deviation (₹11592) of the total cost calculated from observed values.

The code for random generation for the gamma distribution (the rgamma function in R) was used to generate 100000 data points. Also, 123 was set as the seed (set.seed(123)) to ensure reproducibility of the results. The software generated 100000 simulated data points from the gamma distribution with the calculated shape and rate parameters. The lower limit of the total cost, depending upon the actual observed value, was applied to simulated data. By using the pmax function, any value less than the limit was replaced with the applied lower limit. Simulated data points were plotted on a histogram. Summary statistics of mean, median, interquartile range, and credible interval were calculated for simulated data.

Validity testing

Sometimes, random numbers generated with specific seed numbers become correlated. To check for the effects of such correlation, seed numbers were changed, and results were recalculated and compared to find any deviations. For validity testing, both data sources and results from the simulated model were discussed with clinical experts. Analysis from the alternative model (Monte Carlo simulation using uniform distribution) and bootstrapping for the mean were also discussed with the clinical experts. The convergence of the mean and median with an increasing number of simulations from 10000 to 100000 was checked.

## Results

The study included a total of 109 participants, with a near-even gender distribution: 56 males and 53 females. The average age of participants was 37.6 ± 14.3 years, and the average body weight was 65.3 ± 15.9 kg. The most common planned surgery was for calculus cholecystitis with/without choledocholithiasis/cholelithiasis, with 68 cases. Other procedures included hernia repairs (17 cases) and a range of less frequent surgeries, such as for mesenteric cysts, breast cancer, and lipoma of the neck. Among the participants, 14 had co-morbidities: eight had diabetes, five had hypertension, and one had both diabetes and hypertension. Baseline characteristics are represented in Table 1. All the participants of the study underwent general surgical procedures. The types of surgeries, comorbidities, surgical invasiveness grade, and surgical invasiveness index scores are given in Appendix A. All participants of the study were admitted to the surgical ward, and no one required intensive care.

**Table 1:** Summary table of patient variables (n = 109) QR: interquartile range; SD: standard deviation; CV: coefficient of variation; min: minimum; med: median; max: maximum

No.	Variable	Stats/values	Freqs (% of valid)
1	Age (numeric)	Mean (SD): 37.6 (14.3)	46 distinct values
		min ≤ med ≤ max:	
		8 ≤ 37 ≤ 63	
		IQR (CV): 19 (0.4)	
2	Gender (character)	Female	53 (48.6%)
		Male	56 (51.4%)
3	Weight (kg) (numeric)	Mean (SD): 65.3 (15.9)	29 distinct values
		min ≤ med ≤ max:	
		23 ≤ 67 ≤ 98	
		IQR (CV): 21 (0.2)	
4	Duration of stay (days) (numeric)	Mean (SD): 6.5 (2.6)	5: 52 (47.7%)
		min ≤ med ≤ max:	6: 17 (15.6%)
		4 ≤ 5 ≤ 15	7: 13 (11.9%)
		IQR (CV): 2 (0.4)	Others < 10%
5	Total no. of drugs during hospital stay (numeric)	Mean (SD): 8.5 (1.7)	7: 57 (52.3%)
		min ≤ med ≤ max:	10: 27 (24.8%)
		7 ≤ 7 ≤ 11	11: 18 (16.5%)
		IQR (CV): 3 (0.2)	
6	Preoperative RBS (mg/dl) (numeric)	Mean (SD): 115.2 (41)	46 distinct values
		min ≤ med ≤ max:	
		78 ≤ 101 ≤ 325	
		IQR (CV): 14 (0.4)	
7	Duration of surgery (hours) (numeric)	Mean (SD): 1.4 (0.6)	1.00: 53 (48.6%)
		min ≤ med ≤ max:	1.50: 33 (30.3%)
		1 ≤ 1.5 ≤ 3	2.00 and 2.50: 9 (8.3%) each
		IQR (CV): 0.5 (0.4)	

**Figure 1:**
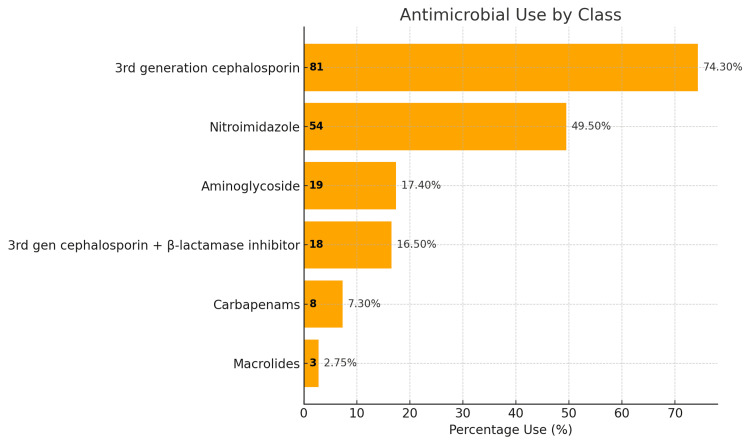
Use of antimicrobials for antibiotic prophylaxis postoperatively (percentage denotes proportion of patients receiving that medication)

The frequency rates of various antimicrobials are given in Figure 1. The most prescribed antimicrobial is the third-generation cephalosporin, accounting for 74.30% (n = 81). This is followed by Nitroimidazole at 49.50% (n = 54). This study highlights a significant preference for third-generation cephalosporins, which indicates their effectiveness or a trend in prescribing practices.

The results of the study on antimicrobial use are summarized in Table 2. The medication distribution for the participants shows a range of treatments administered. The most commonly used medication was Inj. Pantoprazole (91.7%), closely followed by Inj. Emeset and Inj. Tramadol each given to 99 participants (90.8%). Third-generation cephalosporin, Inj. Ceftriaxone was the most commonly prescribed antimicrobial (75.2%), while Inj. Metronidazole was administered to 55 participants (50.4%). Less frequently used were Inj. Clindamycin and Inj. Diclofenac (both 0.91%). Inj. Paracetamol and Inj. Tranexamic acid was given to 47 (43.1%) and 38 participants (34.8%), respectively.

**Table 2:** Frequency of the medications prescribed in the study in descending order

Medication	Number	Percentage
Inj. Pantoprazole	100	91.7
Inj. Emeset	99	90.8
Inj. Tramadol	99	90.8
Inj. Ceftriaxone	82	75.2
Inj. Metronidazole	55	50.4
Inj. Paracetamol	47	43.1
Inj. Tranexamic acid	38	34.8
Inj. Amikacin	18	16.5
Inj. Durataz	17	15.6
Inj. Meropenem	9	8.2
Inj. Ranitidine	9	8.2
Tab. Disperzyme	9	8.2
Inj. Buscopan	7	6.4
Inj. Clindamycin	1	0.91
Inj. Diclofenac	1	0.91

**Figure 2:**
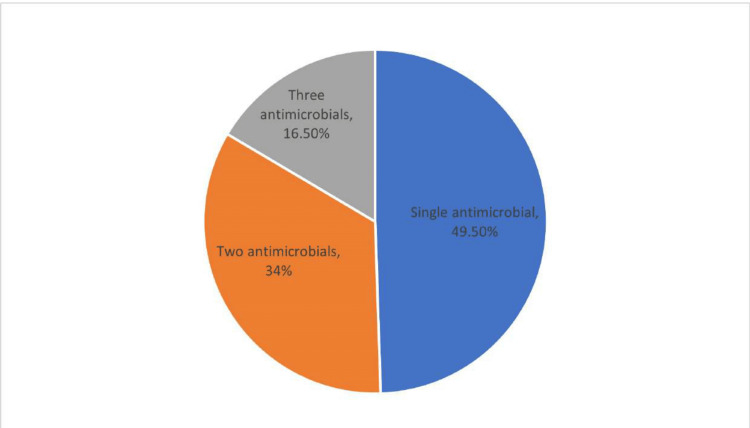
Frequency of the study population receiving one or more than one antimicrobial

The data reveal that the majority of cases (49.50%) involve the administration of a single antimicrobial agent. This is followed by the use of two antimicrobials, which account for 34% of the cases. The least common practice is the use of three antimicrobials, representing 16.50% of the total cases (Figure 2).

Simulation results

**Figure 3:**
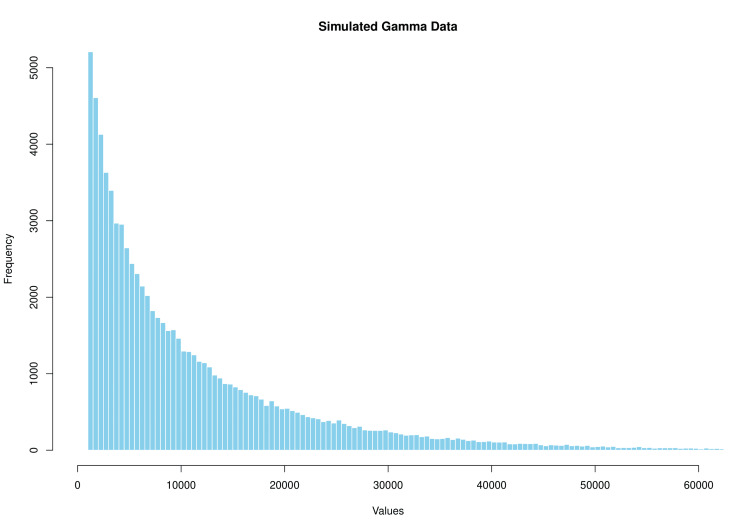
Simulated gamma distribution of postoperative medication costs

The simulation was conducted to model the postoperative medication costs in a tertiary care hospital, with a specified mean cost (₹9175) and standard deviation (₹11592) derived from real data. The simulation involved generating 100000 data points from a gamma distribution using the gamma function in R, representing individual patient costs. The shape and rate parameters were calculated based on the specified mean and standard deviation. The simulated data were then truncated at a minimum value of 1006 using the pmax function to account for the minimum medication cost. Histograms with varying y-axis limits were created to visualize the cost distribution (Figure 3).

The simulation results showed a mean medication cost of ₹9332 (around $111 USD), which is close to the specified mean of 9175. However, the median cost (₹4990, around $60 USD) was significantly lower than the mean, indicating a positively skewed distribution of costs. The interquartile range (IQR) was large at 11103.8, showing substantial variability in patient costs. The 95% credible interval was (1006, 41620.94), providing a wide range of plausible costs. The quantiles also demonstrated the skewed nature of the cost distribution.

**Figure 4:**
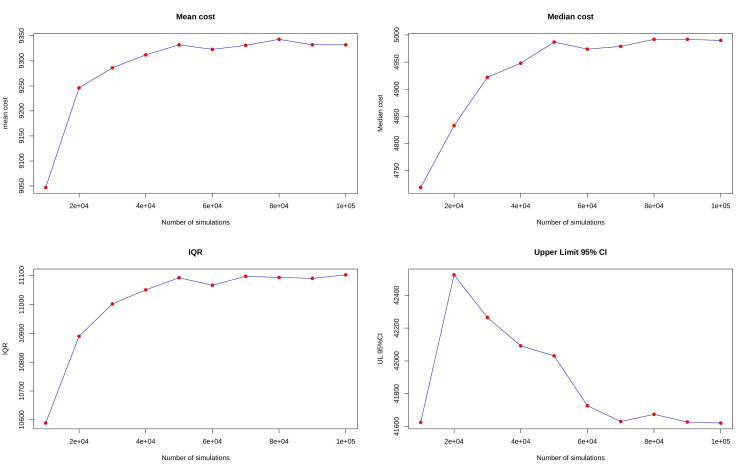
Convergence of simulated cost values with an increase in the number of simulations

The seed number was changed, and the data was reanalyzed. Mean, median, and IQR were found to be 9192, 4996, and 11137, respectively, which were not too different from earlier values. Convergence with an increasing number of simulations was tested, and values for both mean and median appear to converge as the number of simulations increases (Figure 4). The bootstrapped mean was found to be 5541 with a standard deviation of 835 (Appendix A). Total cost was also calculated using a Monte Carlo simulation using uniform distribution, where variability was allowed to propagate on all four types of medication costs (Appendix A).

Comparison with the minimum daily wage

The economic burden of postoperative medications is substantial when compared to India's minimum daily wage. On average, patients would need to work for about 23 days to cover the mean cost of these medications based on the minimum wage for the state of Meghalaya (₹402) as of November 2023 [13]. For the top 2.5% of patients with the highest medication expenses, the financial strain is even more severe, requiring up to 104 days of unskilled labor to meet these costs. This significant disparity underscores the considerable economic pressure that postoperative medication costs place on patients, particularly those with lower incomes.

## Discussion

The primary aim of this study was to evaluate the financial burden of postoperative medication costs for patients undergoing elective surgery at a tertiary care hospital. By utilizing real-world data and simulating a larger patient population, the study offers a comprehensive estimate of these costs' economic impact.

To ensure the validity of our simulation model, we employed several rigorous validation techniques. Discussions with clinical experts confirmed that the model's assumptions and parameters were realistic and aligned with actual clinical practices. Comparisons between raw data, direct estimates, and simulation outcomes demonstrated strong alignment, supporting the model's accuracy. The model's robustness was further validated by the convergence of simulation results with an increasing number of simulations. The gamma distribution, chosen for its suitability in modeling positively skewed data, was particularly effective given the typical cost data distribution, where a small number of cases incur very high costs while most have lower costs. The gamma distribution's definition for positive values only aligns well with cost data, and our simulation defined a lower limit accordingly [17-19].

Our study found that nearly half of the cases preferred single-agent antimicrobial therapy, consistent with guidelines aimed at reducing antibiotic resistance [20]. The prevalent use of third-generation cephalosporins suggests their effectiveness in preventing SSIs due to their broad-spectrum activity against common pathogens. Nitroimidazoles, used for their anaerobic coverage, are also important for surgeries involving the gastrointestinal tract [21]. While these prescribing patterns are essential for reducing SSIs and improving outcomes, they have substantial financial implications, especially for economically disadvantaged patients [22].

The real-world data showed a mean medication cost of ₹9175 with a standard deviation of ₹11592, indicating significant variability. The simulation model produced a slightly higher mean cost of ₹9332, closely aligning with the real-world data. However, the median cost from the simulation was lower at ₹4989.60, with an interquartile range of ₹11103. This suggests that while most patients face moderate costs, a significant minority experience very high expenses. The 95% credible interval, ranging from ₹1006 to ₹41620, further illustrates the wide disparity in potential medication costs. This variability underscores the financial risk for patients, particularly those at the upper end of the cost spectrum, highlighting the need for safety nets like Ayushman Bharat that can accommodate these extremes.

Comparing our findings to global data, the observed financial burden is consistent with international trends, where postoperative medication costs are a significant portion of total treatment expenses [23]. For example, a Brazilian study reported a similar cost burden, with postoperative medication expenses constituting 30% of total healthcare costs, largely due to advanced antibiotics like cephalosporins [24]. In contrast, studies from high-income countries like the United States report lower relative medication costs due to broader insurance coverage and higher average incomes, which reduce out-of-pocket expenses [25].

In India, previous research has shown that postoperative medication costs vary widely based on the type of surgery and hospital prescribing practices [26]. Our findings are consistent with these reports, particularly in the high prescription rate of third-generation cephalosporins. However, our identified cost burden may be higher than in some earlier studies, potentially due to the inclusion of real-world data from a tertiary care setting during the COVID-19 pandemic, which may have influenced prescribing practices.

The financial burden of postoperative medications is substantial, with patients needing an average of 51 workdays to cover costs. For 2.5% of patients, this extends to 104 days, highlighting the disproportionate impact on those with higher expenses. This presents a considerable challenge for low-income families, many of whom may not be covered under government schemes like Ayushman Bharat [27].

While the Ayushman Bharat program aims to provide financial protection, its cost ceiling may leave some patients with substantial out-of-pocket expenses. Our study suggests that a significant number of patients could exceed this ceiling, emphasizing the need for flexibility in the program and timely revisions to the sanctioned treatment amounts.

Several limitations of our study should be noted. The small sample size of 109 patients limits the generalizability of our findings. Additionally, the study was conducted in a public sector teaching hospital, where treatment protocols may differ from private hospitals. The study did not take into consideration potential cost variations by surgery type. The COVID-19 pandemic also potentially influenced the patient population and hospital prescribing practices. Another limitation was that American Society of Anesthesiologists Physical Status (ASA-PS) scores were not recoded in our study, though comorbidity data were collected. Moreover, the gamma distribution's assumptions about data distribution might not fully capture extreme outliers or complex data patterns. Additionally, indirect costs (e.g., hospital stay, lost wages) are not considered. Vendor-specific discounts were ignored from the assessment, which might slightly alter the absolute numbers. However, we believe that the general conclusions of the study are still valid.

## Conclusions

Our study highlights key patterns in antimicrobial use and their financial implications for patients undergoing elective surgeries. Nearly half of the cases received single-agent antimicrobial therapy, aligning with recommended practices to reduce antibiotic resistance. The frequent use of third-generation cephalosporins reflects their effectiveness in preventing SSIs, while nitroimidazoles were commonly used for gastrointestinal procedures due to their anaerobic coverage. Although these prescribing choices support better clinical outcomes, they also result in significant medication costs, particularly impacting economically vulnerable patients.

Real-world data revealed an average medication cost had substantial variability. A simulation model suggests that most patients incur moderate expenses, while a smaller group faces very high costs. The wide credible interval highlights the potential for extreme financial burden in some cases. Further research should compare the cost burden across different hospital settings and explore the long-term financial impact of postoperative medication costs on patients and their families.

## Appendices

Appendix A 

**Table 3:** Types of general surgical procedures included in the study along with their Surgical Invasiveness Index (SII) scores SII Range Interpretation 0-3: low invasiveness - minor, superficial procedures; 4-7: moderate invasiveness - intermediate tissue disruption; 8-12: high invasiveness - major procedures with significant dissection/resection; 13+: very high invasiveness - complex, multi-organ, or extended duration surgeries Surgical Invasiveness Grade - Interpretation Low: minimal tissue disruption, local anesthesia, day-care procedures; moderate: intermediate complexity, general/regional anesthesia; high: major surgery, deep dissection, organ involvement HTN: hypertension

Planned surgery	Number
Calculus cholecystitis	45
Choledocolithiasis	4
Hernia	11
Cholecystitis with inguinal hernia	4
Mesenteric cyst	2
Ca breast	4
Hydrocele	4
Inguinal hernia with varicocele	2
Appendicitis	9
Varicose vein	2
Leg ulcer	1
Lipoma of neck	2
Cholelithiasis	11
Gall bladder microlithiasis	4
Choledocolithiasis with pancreatitis	2
Cholecystitis with choledocolithiasis	2
Total number of patients	109
Co-morbidities	14 (total)
Diabetes	8
HTN	5
Both	1
